# Implementation Science Translation: Program Sustainment for Managing Distress Behavior in Dementia

**DOI:** 10.1093/geroni/igab046.2061

**Published:** 2021-12-17

**Authors:** Kim Curyto, Ann Kolanowski

## Abstract

Distress behaviors in dementia (DBD) are common in nursing home settings, are distressing, and result in poorer outcomes for residents and staff. We present on the implementation of STAR-VA, an interdisciplinary intervention for effective management of DBD in Veterans Health Administration (VA) nursing home settings, called Community Living Centers (CLCs). A primary focus of this symposium is the use of implementation science concepts to improve and sustain evidence-based programs through tailored implementation strategies and key partnerships. Key implementation science concepts from conceptual frameworks, including the Consolidated Framework for Implementation Research (CFIR) and the use of organizational Knowledge Reservoirs (KR) for sustaining new clinical practices, formed the basis of this work. Their application in health care practice will be discussed using STAR-VA as an exemplar. Interdisciplinary CLC staff feedback during STAR-VA implementation and sustainment is presented, including feedback regarding barriers to integrating new program interventions into usual care processes. Mapping key implementation strategies onto reported barriers informed development of implementation tools and strategies designed to guide adaptions tailored to the needs of the residents and frontline staff, increasing the chances of successful sustainment. Finally, we highlight the importance of key leadership partnerships in implementation of evidence-based programs to improve care of residents with DBD and present strategies for developing these partnerships. Discussion will include the importance of using implementation science to implement evidence-based interventions for effective management of DBD and strategies for sustainment of these effective practices into usual care.

